# The Facile Production of *p*-Chloroaniline Facilitated by an Efficient and Chemoselective Metal-Free N/S Co-Doped Carbon Catalyst

**DOI:** 10.3390/ijms25179603

**Published:** 2024-09-04

**Authors:** Juan-José Villora-Picó, Gema Gil-Muñoz, Antonio Sepúlveda-Escribano, M. Mercedes Pastor-Blas

**Affiliations:** Laboratory of Advanced Materials, Department of Inorganic Chemistry, University Materials Institute of Alicante, University of Alicante, P.O. Box 99, E-03080 Alicante, Spain; jj.villora@ua.es (J.-J.V.-P.); gema.gil@ua.es (G.G.-M.); asepul@ua.es (A.S.-E.)

**Keywords:** N/S co-doped carbon, metal-free catalysts, *p*-chloronitrobenzene, *p*-chloroaniline, melamine, cysteine, calcium citrate

## Abstract

The catalytic hydrogenation of the toxic and harmful *p*-chloronitrobenzene to produce the value-added *p*-chloroaniline is an essential reaction for the sustainable chemical industry. Nevertheless, ensuring satisfactory control of its chemoselectivity is a great challenge. In this work, a N/S co-doped metal-free carbon catalyst has been fabricated by using cysteine as a source of C, N, and S. The presence of calcium citrate (porogen agent) in the mixture subjected to pyrolysis provided the carbon with porosity, which permitted us to overcome the issues associated with the loss of heteroatoms during an otherwise necessary activation thermal treatment. Full characterization was carried out and the catalytic performance of the metal-free carbon material was tested in the hydrogenation reaction of *p*-chloronitrobenzene to selectively produce *p*-chloroaniline. Full selectivity was obtained but conversion was highly dependent on the introduction of S due to the synergetic effect of S and N heteroatoms. The N/S co-doped carbon (CYSCIT) exhibits a mesoporous architecture which favors mass transfer and a higher doping level, with more exposed N and S doping atoms which act as catalytic sites for the hydrogenation of *p*-chloronitrobenzene, resulting in enhanced catalytic performance when compared to the N-doped carbon obtained from melamine and calcium citrate (MELCIT) used as a reference.

## 1. Introduction

Aniline (C_6_H_5_NH_2_) and its substituted derivatives such as *p*-chloroaniline [1] are raw chemical materials used as intermediates in the synthesis of commercial products with a high added value (dyes, pigments, rubber processing chemicals, pharmaceuticals) as well as in the production of diphenylamine, phenolics, herbicides, and fungicides [2]. Thanks to its versatility, this chemical compound has substantial industrial and scientific importance. The aniline market, with a volume of 9.9 million tons in 2023, is expected to exhibit a growth rate of 4.7% during 2024–2032, which means that 15.2 million tons will be reached by 2032 [3]. The synthesis of substituted anilines from abundant low-cost chemicals is currently attracting great interest. The synthesis methods of anilines vary depending on the starting materials [4], but the most widely used synthetic route for simple anilines is the nitration of an aromatic compound, such as benzene, toluene, or xylene, followed by the reduction of the corresponding nitroarene [5,6]. On the other hand, chloronitroarenes, widely used in pesticides and herbicides, are found in wastewater and industrial effluents. They represent an environmental issue and a great threat to human health as they are toxic and carcinogenic and can badly affect the nerves, liver, and kidneys [7]. *p*-Chloronitrobenzene is solid at room temperature; however, its high vapor pressure has an inherent risk of inhalation [8]. Therefore, in recent years, a new strategy based on the reduction of the already existing harmful chloronitroarenes to produce value-added chloroanilines has emerged.

Industry demands very often determine the focus of research. The Béchamp reduction of nitrobenzene using iron in HCl was the first technical procedure developed for the synthesis of aniline [9]. Other processes involving the use of Na_2_S_2_O_4_, Fe, Sn, or Zn in NH_4_OH, although widely used in the industry, are not suitable from an environmental point of view as they produce a large amount of waste residues and show poor selectivity [10]. Developing sustainable and environmentally friendly technologies is essential to solve the industrial and environmental problems.

The development of heterogeneous catalysts emerged following the industry requirements for the search of environmentally friendly and economic processes. Nowadays, 85% of the large-scale production of anilines is based on metal-catalyzed nitroarene hydrogenation [5,11] either in gaseous [12] or liquid phases. The latter is preferred when thermally unstable aromatic nitro compounds are involved [13]. Thus, the hydrogenation reaction has become crucial for a sustainable chemical industry. The reason for that is its series of advantages including the use of molecular hydrogen [14,15] to overcome the issues associated with other mild reducing agents, [16] such as sodium borohydrate [17,18,19], which produces sodium borate as a byproduct, or hydrazine hydrate [20], which, although interesting for being capable of generating hydrogen in situ, has toxicity and safe handling issues that limit its industrial application [6]. Moreover, heterogeneous metal catalysts can be easily separated and reused.

Among the different metal catalysts available, noble metals (Pt [17], Pd [21,22], Au [23], Ru [24], Ir [25], Rh [26]) exhibit excellent catalytic activities but usually low selectivity [11]. Additionally, the elevated price of noble metals highlights the need to find a more economical alternative to meet commercialization requirements [16]. As such, Fe [20,27], Co [19,28,29], Ni [18,30], and Cu [31] were considered. However, metal sintering during thermal activation treatments persisted, which resulted in low metal dispersion. Furthermore, leaching issues were not obviated as metal particles easily fell off from the supporting materials, resulting in a decrease in activity [32].

In order to develop a sustainable and environmentally friendly catalytic reduction process, it becomes essential to completely replace metals. For this purpose, research is necessary to develop catalysts with a low environmental impact that can operate in mild conditions while avoiding tedious post-processing steps and recycling issues. Furthermore, the efforts made to increase activity often cause a considerable decrease in selectivity. The control of chemoselectivity when substituted nitrobenzene is used is very challenging, especially in the particular case of *p*-chloronitrobenzene. Both choro and nitro groups are susceptible of being reduced during the catalytic hydrogenation reaction and produce a mixture of chloroaniline and undesired dechlorinated intermediate products [33,34,35,36]. This makes it even more challenging to find an efficient metal-free chemoselective catalyst for the hydrogenation of *p*-chloronitrobenzene. Hydrogen dissociation and activation of the nitro group are key parameters which have to be considered when it comes to the design of active and selective metal-free catalysts suitable for the hydrogenation reaction.

Carbon materials have excellent stability and low cost and have drawn great attention not only as supports [22,37] of metal nanoparticles but also as catalysts by themselves. These represent great metal-free alternatives to overcome the issues associated with metal catalysts. However, it is necessary to break the homogeneous electron density of the carbon matrix to favor the activation of adsorbed hydrogen. The development of novel carbon materials with good catalytic performance as alternatives to the activated carbon widely used as a metal catalyst carrier is therefore the aim of this research work.

Modification of the carbon material’s surface chemistry and textural structure can importantly affect the physicochemical properties which govern the adsorption–desorption and activation of reactants. Carbon can easily be functionalized by heteroatom doping and thus produce a redistribution of the electron density of carbon atoms which are near the heteroatom dopant. The introduction of heteroatoms can also produce defects in the carbon matrix because of the dissimilar atomic sizes with respect to those of carbon, which can cause unequal charge distribution. Furthermore, the doping is long-lasting as a result of the covalent bonding of the heteroatoms to the sp^2^ carbon structure.

Oxygenated groups are considered the most common dopants in carbons. In fact, these species are formed during the pyrolysis treatment of the carbon precursors [38], although they can also be introduced through post-treatment of the carbon with oxidants, such as hydrogen peroxide [39], ammonia [40], or nitric acid [41]. Other functionalities are not formed naturally during the pyrolysis process, but they are no less important. Some heteroatoms are of great interest due to the properties they confer to activated carbons.

N-doped carbons have been extensively used as supports of metal catalysts due to the availability of carbon sources and their excellent catalytic performance [42]. It has been observed that the presence of nitrogen atoms improves carbon and metal catalyst interactions, favoring metal nanoparticle dispersion and preventing the leaching of the metallic active phase [43]. Recently, metal-free N-doped carbon materials for catalysis applications have attracted great interest. As a result of the different electronegativities of C and N, polarization of the C-N bond occurs, which favors its performance in a variety of catalyzed reactions such as hydrogenation reactions. It is widely considered that carbon activity is increased at high N concentrations [44].

Additionally, the doping of carbon with N improves its adsorption properties. There are a wide variety of works in which doped carbon materials are used as adsorbents [45,46,47,48]. It has been reported [49] that narrow micropores (<1 nm) play a crucial role in the adsorption properties of carbon-based adsorbents. Additionally, the more electronegative N atoms attract electrons from the neighboring less electronegative carbon atoms, making them electronically deficient, which favors hydrogen adsorption capacity [40,50]. Furthermore, the introduction of N generates basic sites, improving the adsorption of some gases such as CO_2_ [51]. This basic characteristic is also beneficial for the adsorption of nonpolar or slightly polar molecules such as toluene and other volatile organic compounds (VOCs) [45,52,53].

Although nitrogen doping is the most popular method, there is a growing interest in co-doping with other heteroatoms. Each heteroatom has an electronegativity and an atomic radius dissimilar from those of the carbon atom, which leads to the modification of the electron charge distribution. The sulfur atom is larger, less electronegative, and has a completely different electronic structure to nitrogen. It is believed that the introduction of sulfur into the carbonaceous matrix generates structural defects [54]. In fact, using sulfur instead of replacing the carbon atom within the graphene layers as occurs with nitrogen results in the occupation of the edges or defect zones [55]. Doping with sulfur provides carbon with highly interesting properties for its application in electrocatalysis and in the manufacture of supercapacitors [56]. The improved performance of sulfur-doped carbons has been found to be linked to sulfide, sulfone, and sulfoxide moieties [57,58,59] as well as to the porosity and the hydrophobicity induced by sulfur. This has been reported to increase the catalytic activity for the oxygen reduction reaction (ORR) [38].

The adsorption and catalytic performance of nitrogen-doped carbons have been broadly investigated, but there are scarce studies on N/S co-doped carbons. In addition, the co-doping of two heteroatoms can have a synergistic effect [60,61,62,63,64]. Previous studies [65] have proven that easing the reactants to reach the catalytic sites is crucial to enhance the catalytic activity, so not only the concentration of heteroatoms but also the number of exposed sites is a determining factor. Therefore, carbon textural properties (surface area, pore volume and distribution) are key parameters that need to be controlled during carbon material synthesis to assure an adequate concentration of exposed active sites.

Several methods of preparing N/S co-doped carbons have been reported [66,67,68]. Some of them are based on making the carbon react with gases containing the desired heteroatom at high temperature. However, the use of toxic reactive gases (NH_3_ and HCN for nitrogen doping; H_2_S and SO_2_ for sulfur doping) is not a good option from the point of view of green and sustainable chemistry. Furthermore, the doping obtained is produced mainly at the surface [41,59,69,70,71,72]. Other methods are based on the use of carbon precursors containing the desired heteroatoms [65,73], which are carbonized in an inert gas atmosphere. However, the heteroatom content generally decreases as the carbonization temperature is raised [65,74,75].

Moreover, many as-prepared carbon materials exhibit very low porosity, so they require a further thermal activation treatment aimed to develop the porosity and increase carbon specific surface area. Activation can be carried out either physically or chemically. Physical activation consists of controlled gasification at high temperature (900–1200 °C) in an oxidizing agent stream such as air, carbon dioxide, or steam, which causes the elimination of carbon oxides from the carbon surface, creating porosity [76]. On the other hand, chemical activation is a one-step treatment where carbonization and activation are carried out simultaneously using a chemical oxidizing and dehydrating activating agent such as H_2_SO_4_, ZnCl_2_, H_3_PO_4_, KOH, and NaOH which, once removed, leaves internal pores [77]. Physical activation is preferred over its chemical counterpart for green production as it is a clean treatment, and no secondary waste product is produced. Nevertheless, low carbon yields and poor specific surface are drawbacks associated with physical activation. Furthermore, there is an inherent risk of losing heteroatoms during the thermal activation treatment, which would considerably reduce the doping level [65,73]. It has been reported that N and S heteroatoms are gradually lost as NH_3_ or H_2_S waste gases, which in turn are a source of environmental pollution [60].

Keeping in mind green chemistry perspectives, the focus of this study has been put on finding eco-friendly, safe, and low-cost materials. As described before, N-doped carbon materials have been extensively studied, but N/S co-doped carbon materials are considerably less investigated. The need to thoroughly look into the role of N and S in the modification of carbon properties arises. Some synthesis procedures are experimentally complicated, with several carbonization and activation steps at high temperature. Developing a simple and environmentally friendly method to fabricate N/S co-doped carbons with a hierarchical porous structure which renders the catalytic sites more accessible is the aim of this study. Cysteine is an affordable and safe material, with N and S in its formulation, which makes it an excellent candidate as a carbon precursor with a potentially significant content of sulfur and nitrogen. However, careful control of the pyrolysis experimental conditions [78,79,80] is necessary to prevent heteroatom losses when the existing bonds between the carbon and heteroatoms are broken at high temperatures or extended treatment times. The N-doped carbon material (MELCIT) selected as a reference for this study exhibits low catalytic activity, which is an indication of the relevance of both the precursor content and the carbonization experimental conditions [65,73]. The N/S co-doped carbon material (CYSCIT) has been prepared following the same experimental conditions that provided the poor catalytic activity of the MELCIT carbon material, which will permit us to gain insights into the effects of N/S dual doping. Both cysteine and melamine were mixed with calcium citrate which acted as porogen agent, so no activation treatment was necessary to develop an adequate porous structure. Thus, the issues associated with heteroatom loss during an activation thermal treatment were obviated.

This study will therefore contribute to the design of N/S co-doped carbon materials with a rational perspective for their application in the metal-free catalytic conversion of the harmful *p*-chloronitrobenzene into the valuable *p*-chloroaniline using the benign molecule hydrogen. For this purpose, mild synthesis conditions, exempt from activation thermal treatments, will be employed in this work and extensive characterization of the N-doped and N/S co-doped carbon materials will be performed.

## 2. Results

**Scheme 1:**
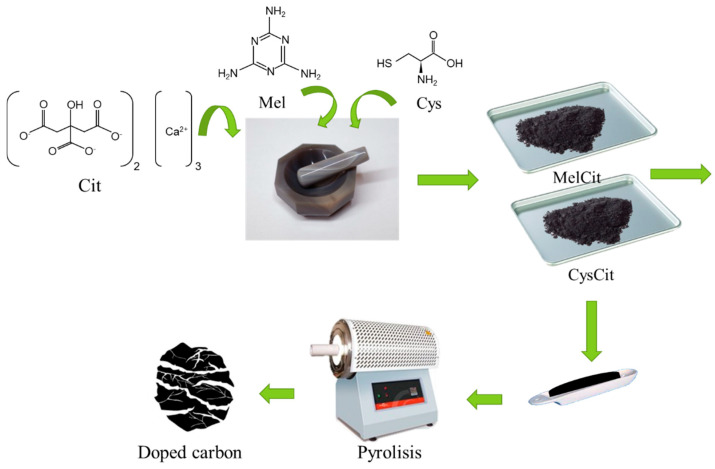
The experimental procedure for the synthesis of doped carbons through the pyrolysis of mixtures of calcium citrate (Cit) with melamine (Mel) or cysteine (Cys).

**Figure 1:**
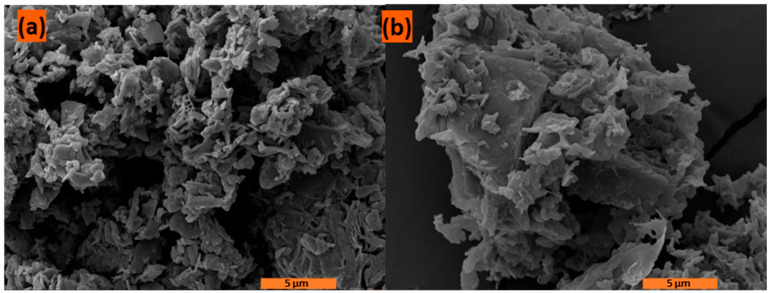
SEM micrographs of (**a**) MELCIT and (**b**) CYSCIT carbons.

Mixtures of calcium citrate with melamine or cysteine were pyrolyzed to obtain the corresponding carbon materials, MELCIT and CYSCIT (Scheme 1). Whereas melamine (C_3_H_6_N_6_) is a source of nitrogen and carbon, cysteine (C_3_H_7_NO_2_S) is a source of nitrogen, sulfur, and carbon. Calcium citrate tetrahydrate Ca_3_(C_6_H_5_O_7_)_2_·4H_2_O is used as porogen in order to develop the porosity without the need of a post-carbonization treatment [65]. Thus, the risk of losing functional heteroatoms from the carbon surface during a thermal activation treatment at high temperature is avoided. The MELCIT and CYSCIT carbons obtained following this procedure showed a similar globular morphology, as observed in the corresponding SEM micrographs (Figure 1).

**Figure 2:**
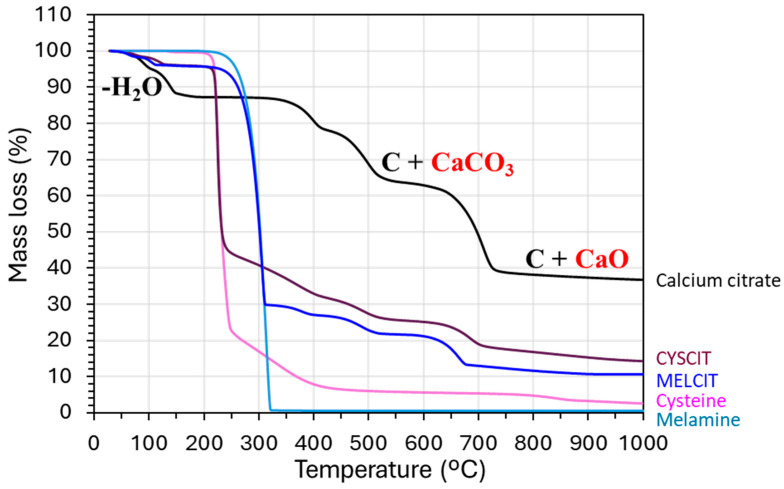
TGA profiles of calcium citrate tetrahydrate, melamine, L-cysteine, and corresponding MELCIT and CYSCIT mixtures. N_2_ atmosphere. Heating rate: 5 °C/min.

**Scheme 2:**
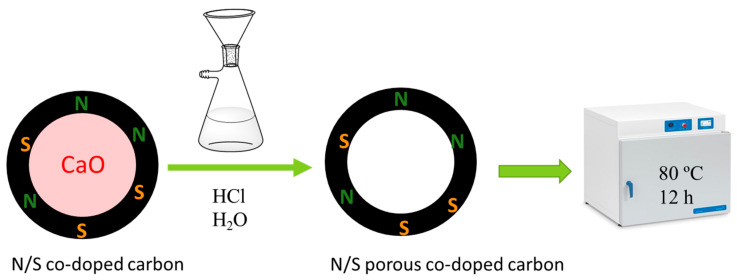
The experimental procedure for the removal of the CaO template.

The decomposition of calcium citrate tetrahydrate, melamine, L-cysteine, and their mixtures was followed up by thermogravimetric analysis. The thermogram of calcium citrate tetrahydrate shows several loss steps (Figure 2). First, dehydration is produced in two successive steps at 60–100 and 100–150 °C, each involving the release of two water molecules [81]. Between 300 and 550 °C, calcium citrate decomposes, producing a calcium aconitate intermediate that evolves into calcium carbonate. Beyond 600 °C, the decomposition of calcium carbonate into carbon monoxide and carbon dioxide is produced, which leaves a residue formed by calcium oxide and carbon [82,83]. During the heat treatment, melamine decomposes into different reactive nitrogen (g-C_3_N_4_) [51,84,85,86] and carbon nitride gases such as C_2_N_2_^+^, C_3_N_2_^+^, and C_3_N_3_^+^ [87] and cysteine decomposes into sulfur-containing carbon species (g-S-C_3_N_4_) [75,88,89]. Under the experimental conditions used in this work, complete decomposition of melamine occurs at around 330 °C, whereas cysteine decomposition leaves a residue of ~3% at 1000 °C. Therefore, when the mixtures are submitted to a pyrolysis treatment at 800 °C, several processes take place: the decomposition of calcium citrate, the evolution of CO and CO_2_, the formation of CaO [83], and the incorporation in the carbon lattice of N and S atoms present in the N/S-containing gaseous species. This incorporation can proceed via direct substitution of C atoms with N/S heteroatoms or by the reaction with the oxygen functionalities in the carbonization intermediates produced at each temperature. During this process, CaO will act as a template of the carbonization products. The effective removal of CaO by an acid treatment wash is necessary to leave a well-developed porous structure in the N/S co-doped carbon (Scheme 2).

Characterization of the carbon materials by CHNS and XPS will provide information about their bulk and surface chemical composition, respectively, whereas adsorption measurements will allow us to evaluate their surface porosity.

Similar percentages of nitrogen are detected in both carbons by CHNS elemental analysis (Table 1) and XPS (Table 2), although a considerably lower percentage of sulfur is detected on the surface of the CYSTIT carbon (1.75 at. %) when compared to the bulk (7.61%). It must be noted that the larger S atom is more difficult to incorporate in the carbon lattice, whereas the N atom, with a similar size to the C atom, can easily substitute a C atom in the carbon framework.

Likewise, Ca is detected by XPS from the remains of calcium oxide. During the pyrolysis treatment, the largest Ca is not incorporated in the carbon lattice and stays as CaO [83,90]. The removal of CaO using the HCl treatment was not totally effective in the case of the MELCIT carbon, which also exhibits a higher percentage of O. This might be produced by the entrapping of CaO in the MELCIT carbon porous structure, making its removal more difficult compared to the CYSCIT carbon.

Oxidation state and chemical environment information have been extracted from the XPS curve fitting of the high-resolution spectra of each element. The different carbon species have typical C 1s binding energies: C-C centered at 284.60 eV, C-O at 285.80 eV, C=O at 287.20 eV, and O-C=O centered at 288.85 eV [91]. The relative amounts of the different species are summarized in Tables S1–S3 in the Supplementary Materials. C-N and C-S binding energies are similar to those of C-O, so more accurate information about N and S functionalities can be extracted from N 1s and S 2p high-resolution spectra.

**Figure 3:**
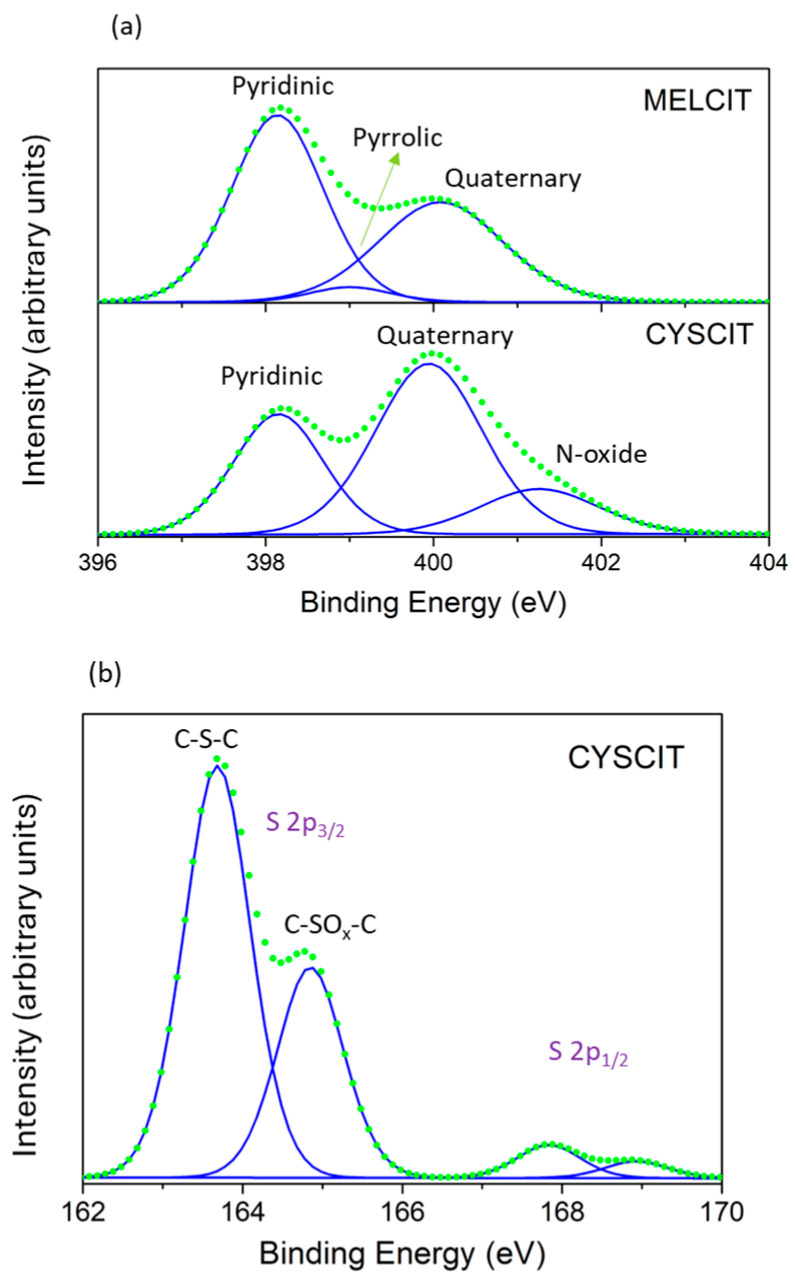
The curve fit of the (**a**) N 1s (**b**) S 2p high-resolution XPS spectra of the carbons.

**Scheme 3:**
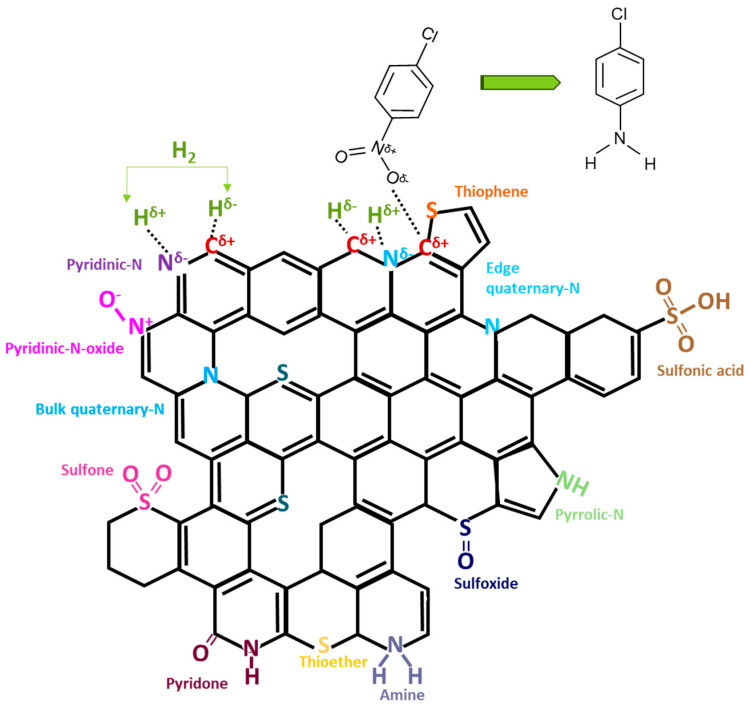
A schematic diagram showing the different possible N and S functionalities in a N/S co-doped carbon and a possible mechanism of the hydrogenation of *p*-chloronitrobenzene activated by N-C-S functionalities.

Several N species can be identified from their typical binding energies in the N 1s high-resolution spectra: pyridinic nitrogen centered at 398.15 eV, pyrrolic nitrogen centered at 399.10 eV, quaternary nitrogen—also called graphitic nitrogen—centered at 400.10 eV, and oxidized nitrogen centered at 401.30 eV [91,92,93]. In the case of the N-doped carbon prepared from a mixture of melamine and calcium citrate (MELCIT), mainly pyridinic (56%) and quaternary nitrogen (40%) are present and some contribution of pyrrolic nitrogen (4%) is detected (Figure 3a and Table S2). However, the N/S co-doped carbon obtained from cysteine and calcium citrate (CYSCIT) shows a more important contribution of quaternary N (53%) compared to the pyridinic N contribution (31%) and some presence of oxidized nitrogen species (16%) at higher binding energies. In addition, the deconvolution of the S 2p peak of CYSCIT shows the incorporation of sulfur into the structure (Figure 3b and Table S3) mainly in the form of C-S-C species (2p_3/2_ at 163.68 eV, 2p_1/2_ at 166.8), although a slight percentage (7%) of oxidized -C-SO_x_-C species (2p_3/2_ at 167.83 eV, 2p_1/2_ at 169.0) is also detected. This suggests that S introduction is produced next to quaternary N or pyridinic N, both of them located preferably at the edges or defective sites where S is mainly inserted (Scheme 3).

**Figure 4:**
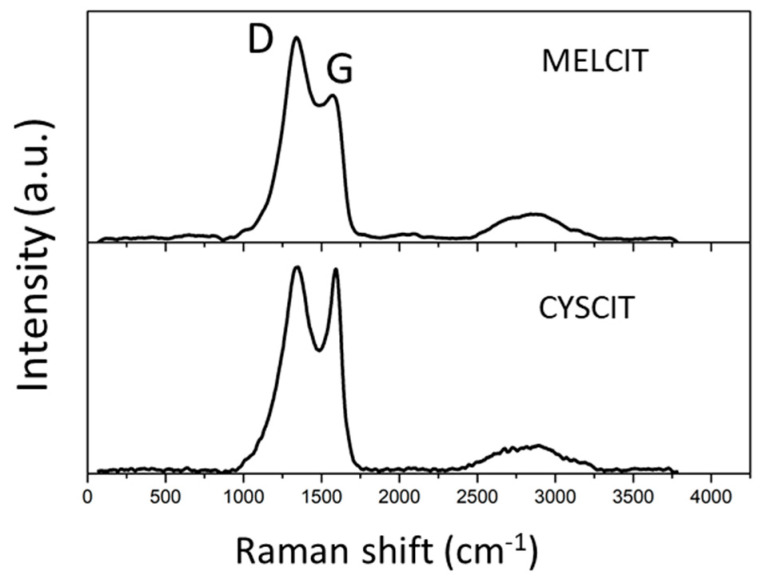
Raman spectra of carbons obtained from mixtures of calcium citrate with melamine (MELCIT) or cysteine (CYSCIT).

The degree of crystallinity in the microstructure of the carbons was assessed by Raman spectroscopy (Figure 4). The G band located around 1580 cm^−1^ is the primary mode in graphite and results from the E_2g_ symmetry of the sp^2^ bonds’ in-plane-bending vibration. G stands for graphite and this band denotes crystallinity. On the other hand, the D band located around 1355 cm^−1^ is the disorder band. It results from the A_1_’ symmetry of the sp^2^ carbon atoms’ breathing mode vibration and is characteristic of amorphous carbon. Therefore, the I_D_/I_G_ ratio serves as an indication of the ordering degree of the carbon matrix; a higher I_D_/I_G_ ratio reflects a more disordered carbon structure with the presence of defects [94,95,96,97].

The carbon obtained from melamine and calcium citrate (MELCIT) shows the highest I_D_/I_G_ ratio (1.41) (Table 3). It must be considered that the XPS analysis of this sample (Table 2) revealed the presence of Ca (1.57 at. %) from calcium oxide produced during the pyrolysis treatment. Ca size (2.31 Å) is significantly larger than that of C (0.70 Å), N (0.65 Å), and S (1 Å). Thus, Ca cannot substitute a C atom in the carbon lattice but produces the disruption of some of the C-C linkages, resulting in a decrease in the ordering [83]. However, in the carbon obtained from cysteine and calcium citrate (CYSCIT), more effective removal of calcium oxide during the washing experimental procedure with HCl is achieved. This results in a more ordered structure; so, an I_D_/I_G_ ratio of 1.02 is calculated from the corresponding Raman spectrum.

**Figure 5:**
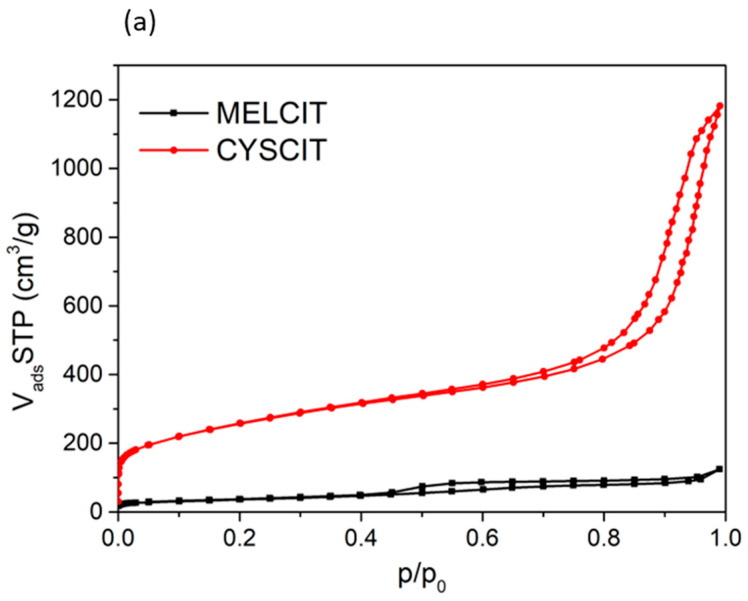

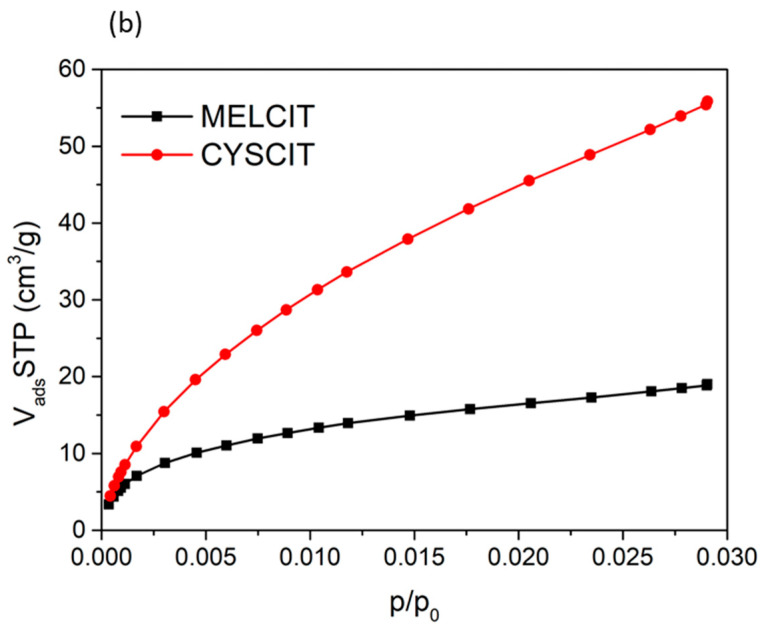
(**a**) N_2_ adsorption–desorption isotherms (−196 °C); (**b**) CO_2_ adsorption isotherms at 0 °C of MELCIT and CYSCIT carbons.

The carbon porous structure was assessed by N_2_ physical adsorption at −196 °C and CO_2_ physical adsorption at 0 °C (Figure 5) and the corresponding textural parameters were calculated (Table 4 and Table 5). The carbons obtained from the mixtures of calcium citrate with melamine (MELCIT) or with cysteine (CYSCIT) show quite different N_2_ adsorption isotherms (Figure 5a). The MELCIT carbon adsorbs very little throughout the range of relative pressures, while the CYSCIT carbon shows an adsorption isotherm intermediate between type Ia, with adsorption at low relative pressures, which is an indication of the presence of micropores, and type IVa, with a pronounced slope at high relative pressures, which denotes the presence of mesopores. Both adsorption–desorption isotherms have hysteresis cycles, produced by capillary condensation in the pores. However, the hysteresis cycles are somewhat different. The MELCIT carbon shows an H4-type hysteresis loop due to narrow slit-shaped pores, where CaO might presumably be retained, while CYSCIT has an H1-type hysteresis loop, due to “ink-bottle”-shaped pores. These kinds of pores can be either cylindrical with both ends opened or can show a narrow short neck [98]. The textural data obtained from the N_2_ adsorption–desorption isotherms shown in Table 4 reveal that the MELCIT carbon has a low porosity and a considerably lower specific surface area (V_total_ = 0.15 cm^3^/g and S_BET_ = 126 m^2^/g) than the CYSCIT carbon (V_total_ = 1.38 cm^3^/g and S_BET_ = 856 m^2^/g).

The narrow microporosity was more exhaustively studied by CO_2_ adsorption measurements at 0 °C (Figure 5b) and by the application of the Dubining–Raduschkevich (D-R) model, which allowed us to determine V_micro_ (CO_2_) and compare it to the volume of the micropores obtained by N_2_ adsorption measurements at −196 °C, i.e., V_micro_ (N_2_). CO_2_ and N_2_ molecules have similar critical dimensions (0.28 nm and 0.30 nm, respectively), but at 0 °C, the diffusion of CO_2_ molecules through the narrow micropores is enhanced, whereas the diffusion of nitrogen molecules is hindered at −196 °C. Therefore, values of V_micro_ (CO_2_) larger than V_micro_ (N_2_) are an indication of a very narrow porosity, where equilibrium is harder to achieve during nitrogen adsorption at –196 °C. On the contrary, if V_micro_ (CO_2_) results in being smaller than V_micro_ (N_2_), this means that a broader range of micropores is present.

Considerable differences in CO_2_ adsorption of the MELCIT and CYSCIT carbons are observed (Figure 5b). The quantification of the textural parameters (Table 5) shows that Vmicro (CO_2_) > Vmicro (N_2_) in MELCIT, which is an indication of the existence of very small micropores, in which the N_2_ molecule is not able to penetrate, whereas Vmicro (CO_2_) < Vmicro (N_2_) in CYSCIT is an indication of the presence of micropores with a broader size distribution.

The analysis of the adsorption data confirmed that the introduction of S into the carbon matrix contributes to form a hierarchically porous structure formed by mesopores and wide micropores. This results in an increased surface area, which renders additional accessible sites. Therefore, it is expected that the adsorption capacity and the catalytic performance are improved.

**Figure 6:**
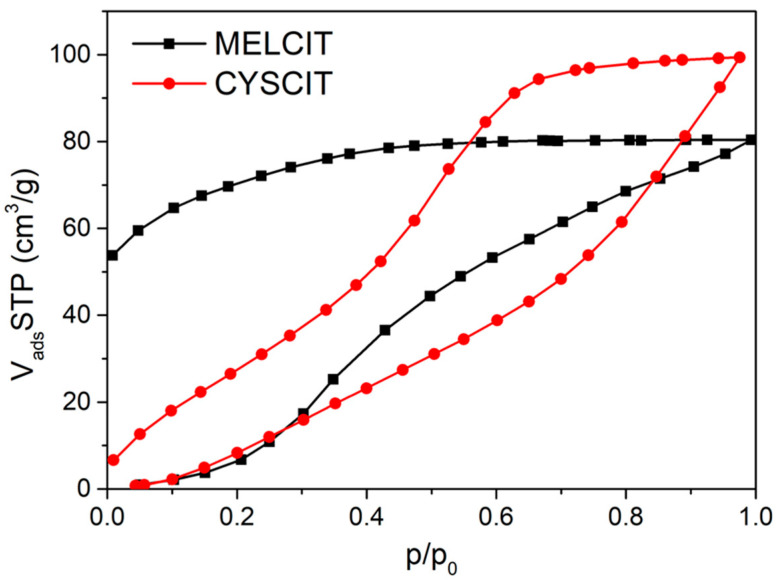
H_2_O adsorption–desorption isotherms at 25 °C of MELCIT and CYSCIT carbons.

The carbon surface is inherently hydrophobic; however, its functionalization with heteroatoms can enhance water adsorption as the functionalized sites can act as anchoring sites for water even at low humidity levels [69]. This could be a drawback or a beneficial feature depending on the carbon specific application. Regardless of the desired hydrophobic/hydrophilic nature of the tailored carbon surface, the water adsorption capacity at low relative pressures (low degree of humidity) is indicative of the quantity of surface functional groups. On the contrary, adsorption at high pressures is an indication of the development of porosity, as a high pore volume enhances the growing of water clusters [45,48]. Therefore, water adsorption–desorption experiments at 25 °C were carried out. As can be observed (Figure 6), water adsorption–desorption isotherms do not become too close. This may be due to the presence of calcium oxide produced from calcium citrate in the carbon lattice, which causes a strong interaction between surface hydroxyl groups and water molecules, preventing their desorption. This is more evident in the MELCIT carbon, which showed 1.57 at. % of Ca determined by XPS measurements.

The performance of the MELCIT and CYSCIT doped carbons as metal-free catalysts in the hydrogenation of *p*-chloronitrobenzene was tested. It should be highlighted the fact that these carbons were not subjected to a thermal activation treatment after carbonization of the precursors, so their activity and selectivity in the reaction may be determined by the carbon surface chemistry (determined by the introduction of N and or S heteroatoms) and porosity (imparted only by the presence of calcium citrate in the precursor mixture). Both carbons showed 100% selectivity for nitro group hydrogenation to produce the corresponding chloroaniline. It is worth mentioning that it is quite challenging to selectively hydrogenate the nitro group when other potentially reducible groups such as halides are in the same nitroarene molecule. This requires the use of a chemoselective catalyst [99]. For this reason, a particular halonitroarene compound, *p*-chloronitrobencene, was selected for this study. The experimental results show that full selectivity was achieved with both MELCIT and CYSCIT, and intermediate compounds, such as 1-chloro-4-nitrosobenzene and 4,4′-dichloroazoxybenzene, found in the presence of metal catalysts [29] were not detected in any case (Figures S1 and S2).

However, the MELCIT and CYSCIT carbon materials showed quite different catalytic performances in the hydrogenation of *p*-chloronitrobencene. Carbon functionalization produced by the introduction of the more electronegative N atom and the larger S atom produces a redistribution of the electronic charge of C atoms next to N and S atoms and also induces the formation of defective sites in the carbon lattice. This affects the adsorption of the hydrogen molecule after its heterolytic dissociation into H^δ+^ and H^δ−^ and also affects the adsorption of the chloronitroarene through its nitro group (NO_2_).

**Figure 7:**
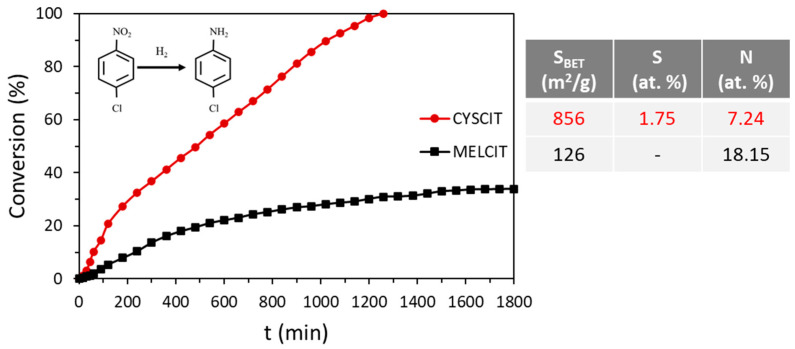
The conversion of the hydrogenation of *p*-chloronitrobenzene in the presence of the MELCIT and CYSCIT carbons.

The MELCIT carbon, obtained from the pyrolysis of a mixture of calcium citrate and melamine (source of N), produces a considerably lower degree of catalytic conversion (Figure 7) compared to the CYSCIT carbon obtained from calcium citrate and cysteine (source of N and S), even though MELCIT has a considerable higher N content (18.15% at.) than CYSCIT (7.24% at.), as determined by XPS. The improved catalytic activity of some N-doped carbons has been widely attributed to quaternary N [38,65,99]. However, some controversial results are reported in the literature, and some authors attribute the catalytic performance to the presence of pyrrolic N functionalities [32,65,73]. XPS analyses showed not only a more important contribution of quaternary N in the CYSCIT carbon, but also the incorporation of the S atom to the carbon matrix. Therefore, the enhanced catalytic performance of the CYSCIT carbon in the hydrogenation reaction must be determined by the synergistic effect produced by the presence of S and N heteroatoms, together with the well-developed porosity and large specific surface provided by the evolution of CO and CO_2_ during calcium citrate decomposition during pyrolysis. The effective removal of Ca atoms is crucial. Otherwise, the MELCIT carbon, even though it has a higher N content, shows entrapped CaO and has a considerably lower specific surface area and pore volume. This negatively affects its catalytic performance.

**Figure 8:**
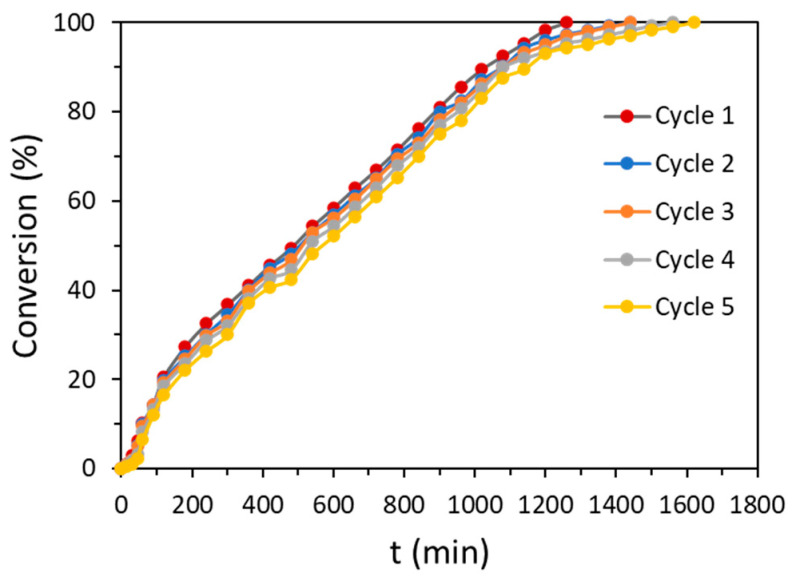
The reusability of the CYSCIT carbon in the hydrogenation of *p*-chloronitrobenzene.

The reusability of the CISCYT carbon catalyst was studied under the same reaction conditions during five consecutive cycles. After each cycle, the used catalyst was recovered by filtration, washed with ethanol, and subjected to a drying process in an oven for 6 h at 80 °C, prior to its reuse. Figure 8 shows that there is a gradual increase in the time required to achieve complete conversion after each cycle due to partial deactivation of the catalyst by the decrease in the number of actives sites. However, the selectivity was not affected and the catalysts were fully selective to *p*-chloroaniline.

## 3. Discussion

The performance of the carbon materials is determined by their surface chemistry, structure, and porosity, so the interpretation of the experimental results is not an easy task. The presence of defects produced by heteroatom doping is also relevant. The larger electronegativity of N (electronegativity: 3.04) with respect to C atoms (electronegativity: 2.55) results in a positive charge density on the adjacent C atoms [100]. It has been observed that this may result in enhanced O_2_ adsorption in oxide reduction reactions (ORRs) [60,80,101,102,103,104] and CO_2_ adsorption in CO_2_ reduction to CO (CO_2_RR) [105]. Other carbon materials doped with the low-electronegative atoms such as P (electronegativity: 2.19) [106] and B (electronegativity: 2.04) [107] have also shown relevant catalytic activity for ORR [42,108,109]. However, the application of metal-free doped carbons as catalysts in hydrogenation reactions has been much less studied [110].

With respect to the doping with S, it must be considered that the S atom (1 Å), being considerably larger than the C atom (0.70 Å), may introduce strains and stress during its incorporation in the carbon lattice due to steric resistance. This is the reason why S is more readily inserted at the edges or defective sites. It has been measured that S atoms stand at a slightly higher level than the carbon layer plane (approximately 1.18 Å in graphene layers) [111,112]. The defects produced by the introduction of S can act as active sites, enhancing the adsorption of reactants on the carbon surface.

This can also affect the carbon lattice’s electronic properties. S and C electronegativities are similar (S electronegativity: 2.58; C electronegativity: 2.55); thus, it is not expected that an important electron charge transfer between S and C atoms takes place. Instead, the greater polarizability of S atoms compared to N and C atoms may produce a spin density redistribution, generating a positive charge on the S atoms, which could act as catalytic centers. This scenario has been considered to explain the improved catalytic activity in ORRs [92,113,114,115]. It has been reported [116,117] that the effective doping of S (1%) in the form of thiophene produced a mismatch between the S and C outermost orbitals, inserting a new energy level of S between the π and π* energy levels of C, thus favoring electronic transitions. Therefore, the improved catalytic activity of carbon materials by S doping can be ascribed either to the catalytic centers located at the defective sites or to the activation of the neighboring carbon atoms, with enhanced charge or spin densities, induced by S species [38].

Research activity on metal-free N/S co-doped carbon materials has mainly been focused on the ORR [113,118], CO_2_RR [75,119], and hydrogen evolution reaction (HER) [120]. Their application in hydrogenation reactions is less explored and recent works have used N/S co-doped carbon materials as supports of noble metal nanoparticles. The defect sites produced by the introduction of heteroatoms can act as nucleation sites for the metal nanoparticles, facilitating their dispersion and stabilization on the carbon support. For instance, Lu et al. [121] observed a strong coordination between Pd and thiophene-S, which played a relevant role in favoring hydrogen spillover and dissociation. They fabricated a tough chemoselective metal catalyst for the hydrogenation of various halonitrobenzenes. However, in this work, the use of metal catalysts has been avoided and the hydrogenation reaction has been carried out in the presence of metal-free carbon materials. Thus, S moieties close to mainly pyridinic and quaternary N moieties in the CYSCIT carbon (Scheme 3) would produce a synergistic effect which results in increased catalytic activity in comparison to the N-doped MELCIT carbon.

It is worth noting that these carbons obtained from the pyrolysis of melamine or cysteine precursors were not submitted to an activation treatment, and that their porosity is due to the evolution of CO and CO_2_ produced by the degradation of calcium citrate during pyrolysis. Therefore, it is important to achieve effective removal of CaO to obtain a well-developed porous structure. In the MELCIT carbon, some Ca is detected by XPS (Table 1). Ca is very electropositive (electronegativity: 1) and much larger (1.76Å) than S, N, and C, so it is not likely to act as a catalytic active site. On the contrary, the presence of the remaining CaO in the carbon matrix has a detrimental effect and prevents pore development.

The mechanism and the active catalytic species in the hydrogenation of *p*-chloronitrobenzene are still under debate and different works arrive at contradictory results. Thus, it is important to analyze the contribution of each functionality in carbon-based metal-free catalysts for the hydrogenation reaction. Breaking the electroneutrality of carbon materials through the delocalization of electrons in the carbon π-orbitals to create active sites for H_2_ activation is a key factor in the hydrogenation reaction. It has been reported [34] that it is possible to change the carbon band’s structure to a metal-like one by heteroatom doping with the aim of increasing the catalytic performance of carbon and making it similar to that of metals. The combination of N and S dopants may have a synergistic effect [64,122]. While N incorporation in the carbon matrix causes significant electron delocalization, making the different N functionalities act as catalytic sites due to an enhanced electron transfer, S atoms, with a similar electronegativity to C atoms but with a larger atomic size, enhance the catalytic activity by introducing structural defects into the carbon lattice. As a result, both H_2_ and the nitro group (-NO_2_) are readily activated by the N-C-S functionalities of the CYSCIT carbon, which act as a bridge between the reactants. The MELCIT carbon lacks the structural defects provided by S doping and depicts a considerably lower surface area and mesoporosity; so, even though selective hydrogenation is produced thanks to the selective adsorption of the NO_2_ group next to the electrodeficient C atoms close to the N dopants, conversion is considerably lower.

It is still under debate whether pyridinic, pyrrolic, or graphitic (also called quaternary N) nitrogen functionalities are the most active N sites. Pyridinic N is in a six-membered ring bonded to two carbon atoms. It is located either on the edge of the ring or close to the defect sites and is sp^2^-hybridized, providing a p-electron to the π system. Pyrrolic N forms sp^3^ hybridization in a five-membered ring and provides two p-electrons to the π system. Graphitic (quaternary) N normally substitutes C atoms in the graphite layer with sp^2^ hybridization either at the edges or the bulk. It is also possible for the oxygenation of pyridinic N to produce more oxidized species [111,123,124,125] (Scheme 3).

Some studies [32] report that pyrrolic N species in doped carbon nanotubes prepared by post-nitridation treatment are responsible for the decent catalytic activity (around 60%) in nitrobenzene hydrogenation, with a selectivity of about 90%. The designed experiments indicated that the defects on these N-doped carbon materials were not the active sites and that the pyrrolic N species were of paramount importance in the catalyzed hydrogenation of nitrobenzene.

Research efforts aimed to tune the electronic density of N-C bonds through the incorporation of another heteroatom have provided enhanced electrocatalytic activity thanks to the synergistic effects produced between both heteroatoms. Pan et al. [75] synthesized N-S-C layers through the pyrolysis of thiourea as N and S sources and citric acid as a C source and they observed that the incorporation of S in N-C favored CO_2_ activation in CO_2_RR, with pyridinic N being close to S as the main active sites. They also showed that the graphitic N, pyridinic N, and thiophene species found in cysteine-derived carbons play a more relevant role in the ORR than other groups [115].

Guo et al. [89] prepared by hydrothermal synthesis Li_4_Ti_5_O_12_ coated with a N/S co-doped carbon. L-cysteine served as a carbon precursor as well as N and S sources. The N/S co-doped material exhibited good electrochemical performance. However, as the heteroatom and carbon sources all came from the same precursor (L-cysteine), it was difficult to ascribe the improvement in the electrochemical performance of the material only to either the carbon layer produced by the carbonization of L-cysteine or to the heteroatom doping which resulted in the formation of g-S-C_3_N_4_ species.

In a previous work [73], N/S co-doped activated carbon with a considerably high surface area (893 m^2^/g) was synthesized from a polypyrrole–polytiophene copolymer. It was concluded that S functionalities acted as preferential adsorption sites for hydrogen and the nitro group in the catalytic hydrogenation of 1-chloro-4-nitrobenzene. However, the need for an activation treatment to develop adequate porosity in the carbon limited the number of heteroatoms remaining after the thermal activation treatment (0.41 at. % of N and 0.94 at. % of S). This was responsible for the low catalytic activity exhibited by this particular carbon material (conversion of nitrobenzene was less than 50%). In a recent study [65], it was demonstrated that the activation treatment could be avoided in a carbon material obtained from melamine thanks to the porosity imparted by the decomposition of calcium citrate into calcium oxide. However, careful control of the experimental conditions was necessary to achieve decent activity of the obtained N-doped carbon.

It is not under debate that the carbon surface chemistry plays a definitive role in the catalytic hydrogenation of *p*-chloronitrobenzene; however, the porosity and textural properties of carbon materials are determining factors and cannot be underestimated. While microporosity provides a larger number of active sites, mass transport loss is reduced in the mesopores, thus favoring the access of H_2_ to the active sites. From this perspective, not only heteroatom doping but also the carbon structure and porosity are key factors in the catalytic activity of carbon materials [126].

The N-doped and N/S co-doped carbon materials that have been compared in this study were prepared using a green, facile, and economically worthy one-step method for their application as metal-free catalysts with superior catalytic selectivity in the hydrogenation of *p*-chloronitrobenzene. The preparation conditions were selected to produce a selective but poorly active N-doped carbon and to study the synergistic effect of S/N co-doping in the carbon. The superior catalytic activity of the N/S co-doped carbon (CYSCIT) compared to the N-doped carbon (MELCIT) can be attributed to the abundant actives sites resulting from the co-doping, the high surface area, and the well-developed mesoporosity produced by the defects resulting mainly from the doping with S and the effective removal of the CaO template.

A mesoporous structure favors mass transfer and permits a higher doping level. This results in more exposed doping atoms that can act as catalytic sites. The obtained results demonstrate that the combination of electronic effects from N/S co-doping and favorable textural properties endow carbon with prominent hydrogenation performance. The CYSCIT carbon exhibits a mesoporous architecture with S-C-N functionalities. Charge redistribution of carbon in these S-C-N moieties, produced by the dissimilarities between C atoms and the S and N dopant atoms, favors the adsorption and activation the nitro group of *p*-chloronitrobenzene and the heterolytic cleavage of the hydrogen molecule. Therefore, the mesoporous structure and the presence of S-C-N functionalities as a result of the co-doping makes the CYSCIT carbon much more active for the hydrogenation of *p*-chloronitrobenzene compared to the MELCIT carbon. This demonstrates the significant role of the sulfur introduced into the carbon matrix. The mechanism of hydrogenation of *p*-chloronitrobenzene on this CYSCIT metal-free carbon catalyst would progress through several steps: (i) the heterolytic dissociation of the H_2_ molecule produces H^δ+^ that adsorbs preferentially to the more electronegative active sites (N^δ−^-H^δ+^) and H^δ−^ which adsorbs to the more electropositive active sites (C^δ+^-H^δ−^); (ii) the adsorption of nitrobenzene is produced preferentially at the defects where S is located; (iii) the selective hydrogenation of the nitro group of *p*-chloronitrobenzene is then produced preferably at the defects (Scheme 3). Therefore, there is a synergistic effect produced by the proximity of the N and S active sites. This results in a considerable better catalytic activity of the N/S co-doped CYSCIT carbon compared to that of the N-doped MELCIT carbon. Therefore, in this work, facile synthesis of a superior metal-free carbon catalyst for the transformation of the harmful *p*-chloronitrobenzene into the value-added *p*-chloroaniline has been carried out and the active role of S doping has been rationalized.

## 4. Materials and Methods

### 4.1. Materials Synthesis

N-doped carbons were prepared using homogeneous physical mixtures of 3 parts of either melamine (C_3_H_6_N_6_, 2,4,6-Triamino-1,3,5-triazine, M_m_ = 126.12 g/mol, Sigma-Aldrich, Madrid, Spain, 99%) or L-Cysteine (HSCH_2_CH(NH_2_)CO_2_H; 2-Amino-3-mercaptopropionic acid; M_m_ = 121.16 g/mol, Sigma-Aldrich, ≥97%) and 1 part of calcium citrate tetrahydrate ([O_2_CCH_2_C(OH)(CO_2_)CH_2_CO_2_]_2_Ca_3_·4H_2_O, M_m_ = 570.49 g/mol, Sigma-Aldrich, 99%). The mixtures (~28 g) were submitted to pyrolysis under flowing N_2_ in a tubular furnace at 800 °C for 1 h. Afterwards, the samples (3–5 g) were treated with a 10 wt% HCl solution (Sigma-Aldrich, ACS reagent), washed with distilled water, and dried at 80 °C for 12 h. The mass of the remaining samples was approximately 2 g.

### 4.2. Material Characterization

Thermogravimetric analysis (TGA) was carried out using a TGA-DSC 2 instrument (Mettler-Toledo, Cornellà de Llobregat, Barcelona, Spain) with alumina crucibles of 70 μL and sample masses between 3 and 4 mg. A stabilization time of 20 min was allowed before starting the experiment. A nitrogen flow of 100 mL/min and a temperature range between 25 and 900 °C at a heating rate of 5 °C/min were used.

Scanning Electron Microscopy (SEM) was performed using a Hitachi S3000N instrument equipped with a Bruker XFlash X-Ray detector 3001. Prior to the analyses, the solvent occluded in the samples was eliminated in a critical point drying instrument (Electron Microscopy Sciences EMS 850), and the samples were metallized with Au in a metallizer/evaporator BALZERS SCD 004 device.

X-ray photoelectron spectroscopy (XPS) was performed using a fully automated K-ALPHA spectrometer (ThermoFisher Scientific) under ultra-high vacuum operation conditions using Mg-K_α_ radiation (1253.6 eV). A twin crystal monochromator provided a focused X-ray spot with a 400 nm diameter and operated at 3 mA and 12 kV. The instrument has a hemispherical alpha analyzer that operates in constant energy mode at a pass energy of 200 eV to obtain the survey spectra (between 0 and 1350 eV) and 50 eV to perform the narrow scans. Subsequent curve fit adjustment was performed to obtain the atomic surface composition.

Elemental analysis (CHNS) was carried out using a microanalyzer equipped with a Micro TruSpec (LECO) detection system. Helium was used as a carrier gas and the combination of infrared and thermal conductivity detectors permitted the simultaneous detection of carbon, hydrogen, nitrogen, and sulfur.

Raman spectroscopy was performed using a Raman Jasco NRS-5100 device. A 532 mn laser and a 600 line/mm slit between 0 and 4000 cm^−1^ were used.

N_2_ adsorption at −196 °C and CO_2_ adsorption at 0 °C were performed using an Autosorb-6 equipment (Quantachrome Instruments, Boynton Beach, FL, USA). H_2_O adsorption experiments at 25 °C were carried out using a Vstar Win device (Quantachrome Instruments). The samples were degassed prior to the experiment in an Autosorb Degasser (Quantachrome Instruments) at 250 °C for 4 h under vacuum.

### 4.3. Catalytic Tests

The catalytic behavior of the prepared carbon materials was studied in the hydrogenation reaction of *p*-chloronitrobenzene (ClC_6_H_4_NO_2_; M_m_ = 157.55 g/mol; 99%, Sigma Aldrich). The experimental procedure was as follows: A 300 mL high-pressure stainless-steel reactor (Biometa, Asturias, Spain) containing 500 mg of the carbon and 100 mL of a 0.1 M solution of *p*-chloronitrobenzene (ClC_6_H_4_NO_2_, Sigma) in ethanol was used. In all cases, 200 µL of octane (CH_3_(CH_2_)_6_CH_3_, Sigma Aldrich, ≥99%) was added as an internal standard. H_2_ (Carburos Metálicos, Barcelona, Spain) at a pressure of 50 bar was used. The heating of the system began with light stirring (10 rpm). When the temperature reached 150 °C, the agitation was set up at 300 rpm. Periodical extraction of 2 mL aliquots from the reactor permitted us to perform gas chromatography and mass spectrometry using a GC/MS device (QP-2010 GC-MS Shimadzu). An HP-5 capillary column with helium as a carrier gas was selected.

The conversion and selectivity were calculated as follows:

$$Conversion\;\left(\%\right)=100-\frac{\left[CNB\right]}{{\left[CNB\right]}_{0}}*100$$

$$Selectivity\;\left(\%\right)=\frac{\left[CA\right]}{{\left[CNB\right]}_{0}-\left[CNB\right]}*100$$

where ${\left[CNB\right]}_{0}$ is the initial concentration of *p*-chloronitrobenzene and $\left[CNB\right]$ and $\left[CA\right]$ are, respectively, the measured concentrations of *p*-chloronitrobenzene and *p*-chloroaniline during the progress of the reaction.

Averages of the conversion data obtained from five tests were calculated and the standard deviation was calculated (less than 2% in all cases). A blank test with no catalyst was carried out and a null conversion was obtained.

## 5. Conclusions

In this work, a metal-free carbon catalyst with an adequate concentration of N and S dopants, lattice defects, and a developed porous structure has been prepared by one-step carbonization of calcium citrate and cysteine, which produces heteroatom-containing active sites in situ. A thermal activation treatment has been avoided for the sake of preventing the removal of the doping heteroatoms. CYSCIT exhibited superior catalytic activity (100% conversion after 20 h) and outstanding chemoselectivity for the reduction of *p*-chloronitrobenzene. Detailed characterization and analysis of the experimental results led to the conclusion that the higher catalytic activity of the N/S co-doped CYSCIT carbon compared to its N-doped counterpart (MELCIT) can be attributed to the synergistic effect of N and S active sites, its high surface area (856 m^2^/g), its mesoporous structure, and the formation of active defect sites. This study has served to clarify the role of N and S active dopants in the catalytic performance of metal-free carbon catalysts. 

## Figures and Tables

**Table 1:** CHNS elemental analysis (%) of the carbons prepared from the melamine–calcium citrate and cysteine–calcium citrate mixtures.

Carbon Sample	C	H	N	S
MELCIT	51.72	1.41	17.85	0.00
CYSCIT	62.45	1.74	6.97	7.61

**Table 2:** The surface atomic percentages (at. %) obtained by XPS on the surface of the carbons prepared from the melamine–calcium citrate and cysteine–calcium citrate mixtures.

Carbon Sample	C 1s	O 1s	S 2p	Ca 2p	N 1s
MELCIT	73.80	6.48	0.00	1.57	18.15
CYSCIT	86.35	4.5	1.75	0.15	7.24

**Table 3:** The relative intensity between the D and G bands in the Raman spectra of the carbons.

Carbon Sample	I_D_/I_G_
MELCIT	1.41
CYSCIT	1.02

**Table 4:** Total pore volume (V_total_), micropore volume (V_micro_), mesopore volume (V_meso_), and BET surface area (S_BET_), calculated from N_2_ adsorption–desorption isotherms (−196 °C) of MELCIT and CYSCIT carbons.

Sample	V_total_ (cm^3^/g)	V_micro_ (cm^3^/g)	V_meso_ (cm^3^/g)	S_BET_ (m^2^/g)
MELCIT	0.15	0.05	0.099	126
CYSCIT	1.38	0.33	1.05	856

**Table 5:** Micropore volume determined by N_2_ and CO_2_ adsorption isotherms of MELCIT and CYSCIT carbons.

Sample	V_micro_ (N_2_) (cm^3^/g)	V_micro_ (CO_2_) (cm^3^/g)
MELCIT	0.05	0.55
CYSCIT	0.33	0.20

## Data Availability

All data generated or analyzed during this study are included in this article.

## Supplementary Materials

The following supporting information can be downloaded at: https://www.mdpi.com/article/10.3390/ijms25179603/s1.
