# Supplementary material for: The Facile Production of p-Chloroaniline Facilitated by an Efficient and Chemoselective Metal-Free N/S Co-Doped Carbon Catalyst
Source: Int J Mol Sci. 2024 Sep 4;25(17):9603. doi: 10.3390/ijms25179603 (PMC11395487; doi:10.3390/ijms25179603)
Supplement: Supplementary file 1 [file ijms-25-09603-s001.zip › ijms-3134206-supplementary.pdf]

International Journal of *Molecular Sciences*

MDPI

Article

The Facile Production of *p*-Chloroaniline Facilitated by an Efficient and Chemoselective Metal-Free N/S Co-Doped Carbon Catalyst

Juan-José Villora-Picó, Gema Gil-Muñoz, Antonio Sepúlveda-Escribano and M. Mercedes Pastor-Blas *

Laboratory of Advanced Materials, Department of Inorganic Chemistry, University Materials Institute of Alicante, University of Alicante, P.O. Box 99, E-03080 Alicante, Spain; jj.villora@ua.es (J.-J.V.-P.); gema.gil@ua.es (G.G.-M.); asepul@ua.es (A.S.-E.)

* Correspondence: mercedes.pastor@ua.es; Tel.: +34-965903400

Supplementary materials

Table S1. Percentages obtained from the C 1s curve fitting (XPS) of the carbon samples.

Carbon sample	Energy (eV)	Species	at. %	%
MELCIT	284.62	C-C; C-H, C=C	43.93	60
	285.80	C-O, C-N	16.70	23
	287.02	C=O; C=N	9.83	13
	288.94	O-C=O	3.28	4
CYSCIT	284.55	C-C; C-H; C=C	63.97	74
	285.83	C-O; C-N; C-S	13.54	16
	287.16	C=O; C=N	7.36	9
	289.13	O-C=O	1.48	2

Table S2. Percentages obtained from the N 1s curve fit (XPS) of the carbon samples.

Carbon sample	Energy (eV)	Species	at. %	%
MELCIT	398.14	pyridinic	10.12	56
	399.10	pyrrolic	0.71	4
	400.08	quaternary	7.32	40
CYSCIT	398.15	pyridinic	2.27	31
	400.04	quaternary	3.84	53
	401.26	oxidized	1.13	16

Table S3. Percentages obtained from the S 2p3/2 curve fit (XPS) of the CYSCIT carbon.

Carbon sample	Energy (eV)	Species	at %	%
CYSCIT	163.68	C-S-C	1.63	93
	167.83	C-SO_x-C	0.12	7

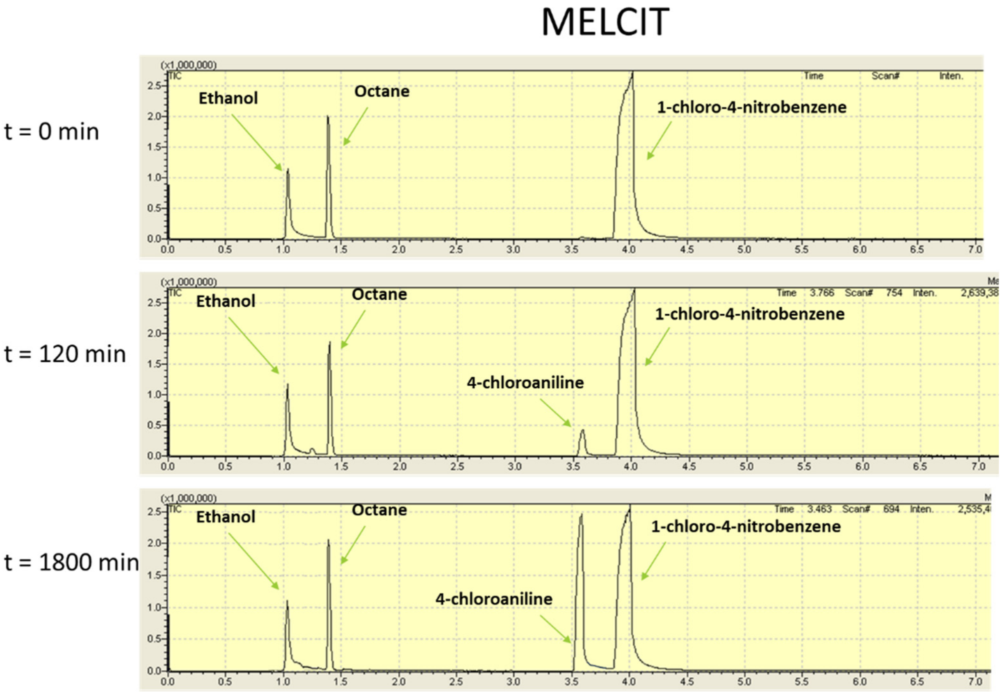

Figure S1. CGC-MS analysis of compounds at different times during the course of the reaction in the presence of MELCIT carbon.

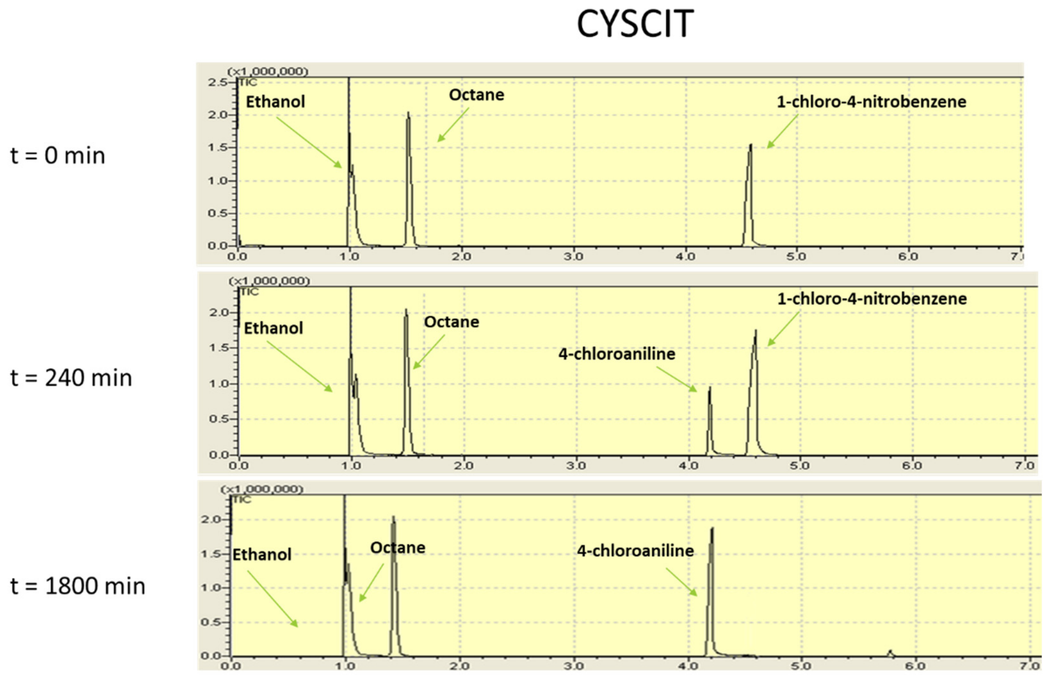

Figure S2. CGC-MS analysis of compounds at different times during the course of the reaction in the presence of CYSCIT carbon.
